# A Bioinspired Stress‐Response Strategy for High‐Speed Soft Grippers

**DOI:** 10.1002/advs.202102539

**Published:** 2021-09-02

**Authors:** Yangqiao Lin, Chao Zhang, Wei Tang, Zhongdong Jiao, Jinrong Wang, Wei Wang, Yiding Zhong, Pingan Zhu, Yu Hu, Huayong Yang, Jun Zou

**Affiliations:** ^1^ State Key Laboratory of Fluid Power and Mechatronic Systems Zhejiang University Hangzhou 310027 China

**Keywords:** bioinspired, high‐speed soft grippers, soft grippers, soft robotics, stress‐response

## Abstract

The stress‐response strategy is one of the nature's greatest developments, enabling animals and plants to respond quickly to environmental stimuli. One example is the stress‐response strategy of the Venus flytrap, which enables such a delicate plant to perceive and prey on insects at an imperceptible speed by their soft terminal lobes. Here, inspired by this unique stress‐response strategy, a soft gripper that aims at the challenges of high‐speed dynamic grasping tasks is presented. The gripper, called high‐speed soft gripper (HSG), is based on two basic design concepts. One is a snap‐through instability that enables the HSG to sense the mechanical stimuli and actuating instantly. The other one is the spider‐inspired pneumatic‐powered control system that makes the trigger process repeatable and controllable. Utilizing the stress‐response strategy, the HSG can accomplish high‐speed sensing and grasping and handle a dynamic grasping task like catching a thrown baseball. Whereas soft machines typically exhibit slow locomotion speed and low manipulation strength for the intrinsic limitations of soft materials, the exploration of the stress‐response strategy in this study can help pave the way for designing a new generation of practical high‐speed soft robots.

## Graphical abstract

Inspired by the stress‐response strategy of the Venus flytrap—combining trigger hairs and bistable lobes to accomplish the predation process, the stress‐response strategy with passive sensing and actuating are applied in a soft gripper, thereby enabling the soft gripper to achieve repeatable high‐speed dynamic gripping as well as controllable trigger sensitivity and force output.

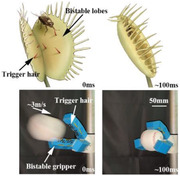

## 1 Introduction

Grasping and manipulating is a major area of interest within the field of robots. In recent years, advances in soft materials science have promoted the development of soft grippers, which excel at grasping unstructured objects with simple sensing and actuating strategies,^[^ ^1^ ^]^ offering a series of cost‐effective solutions for tasks that can rarely be achieved with conventional rigid robotic grippers, including manipulation of delicate objects,^[^ ^2^, ^3^ ^]^ extremely gentle gripping,^[^ ^4^ ^]^ shape adaptive gripping,^[^ ^5^ ^]^ and surface morphology perception.^[^ ^6^ ^]^

Despite these advances, even the most advanced grippers are pale in competition with a human hand, especially for high‐speed dynamic grasping tasks (e.g., catching a thrown baseball). This kind of dynamic grasping task requires a gripper to accomplish perceiving and actuating in a short time, and the soft robotic grippers were limited by two significant shortcomings to accomplish this kind of task. On the one hand, in the terms of sensing, most of the soft robotic grippers rely on user‐provided information instead of embedded sensors. And for the soft robotic grippers with embedded sensors, the speed of these sensors is generally of order 100 ms, limited by the mechanical response of the elastomer.^[^ ^7^ ^]^ On the other hand, in terms of actuating, soft robotic grippers are typically shown small force exertion and slow response time due to the material's softness and structural compliance.

Although the high‐speed dynamic grasping task seems to be an impossible mission for the soft grippers, there are some plants with a soft structure that have the potential to make this mission possible. The predation process of the Venus flytrap is a typical dynamic capture process. When an insect falls on the terminal lobes, the Venus flytrap perceives the movement of the insect by their trigger hairs and closes its terminal lobes to prey on the insect at an almost imperceptible speed. The rapid closure of the terminal lobes is the fastest movement (≈100 ms) in the plant world^[^ ^8^ ^]^ and the secret of producing such a fast movement is the triggerable snap‐through instability to quickly released the energy stored in the terminal lobes under mechanical stimuli.^[^ ^9^ ^]^

Snap‐through instability provides a powerful nonlinear mechanism that triggers rapid response.^[^ ^10^, ^11^, ^12^ ^]^ In recent years, researchers have utilized the snap‐through instability and created a variety of soft machines with fast actuating speeds.^[^ ^13^, ^14^, ^15^, ^16^, ^17^ ^]^ However, high‐speed dynamic grasping tasks require not only fast actuating speed but also fast perception. For robots, instead of over‐reliance on high‐level computation, actuating by mechanical stimuli of the environment is an effective method to achieve rapid perception speed. The delays involved in signal transferring and processing could be eliminated. In 2019, Thuruthel et al.^[^ ^18^ ^]^ presented a bistable gripper that can be triggered by mechanical contact and snap‐through to a closed state with an actuation time of 0.021 s. However, the triggering process is irreversible and requires a manual reset before actuating. In 2020, Partridge et al.^[^ ^19^ ^]^ presented a passive, reflex response unit that uses outside mechanical touch to open and close the valve and inflate the pneumatic network to achieve repeatable mechanical triggering, but a longer response time (about 0.4 s) is required.

**Figure 1:**
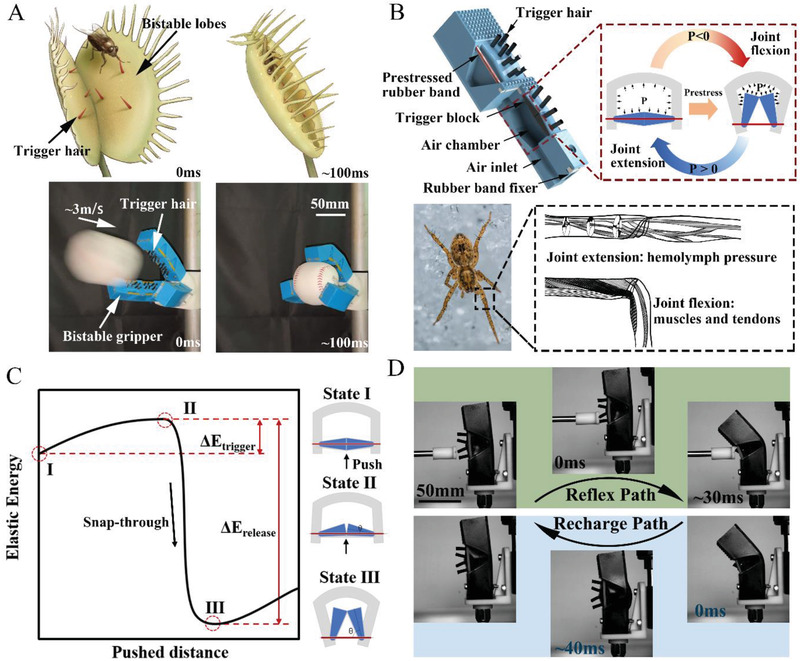
Bioinspired high‐speed soft gripper. A) Inspired by the stress‐response strategy of Venus flytrap's high‐speed predation process, the robotic hand with three HSGs is proposed to realize the dynamic grasping of a thrown baseball. The flying speed of the baseball is about 3 m s^−1^, and this robotic hand can hold it steadily within 0.1 s. B) The schematic design of the HSG and the inspiration of the repeatable trigger structure comes from the joint structure of the spider. Spider's joint extension is driven by hemolymph pressure and joint flexion is driven by muscles and tendons. Similarly, we use rubber bands to imitate muscles and tendons, and air pressure to imitate hemolymph pressure, which is the power source for driving HSG to bend and straighten. Schematics of the joint extension and flexion are adapted with permission from ref. [20] Copyright 1985, Springer Nature. C) Schematic of the elastic energy landscape of the one‐joint HSG shown in D) while being triggered by external pushing. The schematic shows one peak (unstable state II) and two localized minimum energy states (stable states I and III). Stable state I has higher elastic energy than stable state III, which provides the possibility of rapid response. Insets show the joint structure of the HSG at each state. D) The reflex and recharge path of the one‐joint HSG. When an external object touches the trigger hairs, the HSG can accomplish the transition from straightened to bending state within 30 ms. When air pressure is applied to the chamber of the HSG, the HSG can be straightened within 40 ms.

Here, we report a high‐speed soft gripper (HSG) that aims to provide a design paradigm of soft robots inspired by the stress‐response strategy. This gripper is capable of high‐speed dynamic gripping tasks, such as grabbing a thrown baseball (as shown in **Figure** **1**A; and Movie S1, Supporting Information). When a flying baseball hits the trigger hairs of the gripper, just like a fly hits the trigger hairs of the Venus flytrap, it will trigger the bistable structure and quickly release the potential energy to enter another gripping state. This grasping process is completely passive and requires neither high‐level sensing and computing units, nor high‐performance actuators. Besides, taking advantage of bistable characteristics, the gripper can maintain the gripping state without continuous input of external energy.

To allow the HSG to perform gripping repeatable and controllable, a pneumatic control system inspired by the spider joint acting mechanism is designed for it. The main joints of spiders’ walking legs (i.e., the femur‐patella and tibia‐metatarsus joint) are almost exclusively filled with flexor muscles which may be adaptive concerning prey capture.^[^ ^21^, ^22^ ^]^ Instead of extension muscles, the extension of these leg joints is based mainly on an increase of prosomal hemolymph pressure.^[^ ^23^ ^]^ As shown in Figure 1B, the HSG adopt the same strategy, using prestretched rubber bands as the contracting muscles and tendons, and pneumatic pressure as hemolymph pressure. Furthermore, by switching between positive and negative pressure, air pressure can act on the both processes of joint extension and flextion. Utilizing the spider‐inspired pneumatic‐powered control system, the HSG can 1) recover from the state after the trigger, 2) control the trigger sensitivity, 3) generate actively trigger actions without external stimuli, and 4) adjust the gripping force. We demonstrate that the HSG can be readily integrated with the existing robotic platform, and it is more practical for industrial applications than prototypes from previous research.^[^ ^18^ ^]^ This high‐speed responsiveness and repeatable passive grasping ability can enrich the grasping modes of the robot, making the cooperation of the machine gripper and the robot arm much simpler and more inexpensive.

## 2 Results

### 2.1 Design of Repeatable Trigger Structure

Figure 1B illustrates the schematic structure of a double‐joint HSG. The main part of the HSG (blue part in Figure 1B) is made of polyurethane material. Each joint of the HSG is fabricated with an air chamber, and each air chamber is embedded with two trigger blocks (the black part in Figure 1B) which also are made of polyurethane. The bottoms of the two trigger blocks are in contact with each other, and each trigger block has 4 hairs protruding outside the air chamber to perceive mechanical stimuli from different directions. The trigger hairs are stiffer than the main part to transmit contact force to cause the triggering process. Two sets of rubber bands (red part in Figure 1B) on both sides of each joint are hooked to the fixers (white parts in Figure 1B). Figure S1 (Supporting Information) shows the photographs of all parts required to assemble the HSG.

The HSG has an uneven bistable energy structure to actuate rapidly. While being triggered by external stimuli, the HSG transfer from the stable state with higher elastic energy to another stable state with lower elastic energy and release elastic potential energy and convert it into kinetic energy and achieve fast grasping speed. Between the two stable states, an unstable state with local maximum potential energy is required to prevent spontaneous transformation. We designed the structure of the HSG based on this energy principle. We use rubber bands as a cheap and commercially available storage element for elastic potential energy. The elementary model of the elastic energy during the trigger process is shown in Figure 1C. When the HSG is in the straightened state, the trigger blocks inside it get stuck, and the rubber bands are kept elongated, remaining the HSG in a higher energy state (stable state I). When external objects touch the trigger hairs of the HSG, the two trigger blocks will deform and rotate, stretching the rubber bands and deforming the HSG, thus increases the potential energy in the HSG before reaching the unstable state II. While the pushing distance of the HSG keeps increasing, the trigger blocks can no longer maintain a stable stuck station, the entire structure becomes unstable, and the energy stored in the rubber bands is quickly released, and the joint will bend rapidly, and reach the stable state III. The triggering process is shown in Figure 1D as the reflex path. During the triggering process, the energy of state II is slightly lower than the elastic potential energy of state II. The elastic energy difference between the state I and state II is an energy barrier to ensure that the HSG can remain in a straightened state without external stimuli. The elastic potential energy of state III is much lower than that of the second state, which realizes the rapid release of the elastic potential energy and enables the HSG to complete rapid bending.

By inflating the air chamber of the HSG, the air pressure causes the joint to be straightened, and the trigger blocks can be restored to the stuck state. Then the HSG can be maintained in a straightened state again and get ready for the next trigger and grip. The recharging process is shown in Figure 1D as the recharge path.

### 2.2 Control of Trigger Sensitivity

While triggering, external stimuli provide energy to the HSG to cross the energy barrier between state I and state II (denoted as Δ*E*_trigger_ in Figure 1C). With a smaller energy barrier, the HSG might be unexpectedly triggered by external disturbances, while with a higher energy barrier, it might be insensitive to perceive external stimuli. In this section, we focus on how to adjust the design parameters of the HSG to control the sensitivity of the HSG.

The sensitivity of HSG can be quantitatively characterized by the thrust force and distance of triggering. To predict the sensitivity of the HSG under external stimuli, we conducted finite element simulations using a push block to trigger the HSG (as shown in Movie S2, Supporting Information). The HSG model was modeled with dimensions, material properties, boundary conditions, and loads equal to the HSG used later in the experimental validation. The results of the finite element simulations were used to analyze how critical design parameters affected forces and displacements required to trigger the HSG, namely the bottom length of the trigger block, the air pressure applied to the inner chamber, and the number of rubber bands used in each side of the joint were varied.

**Figure 2:**
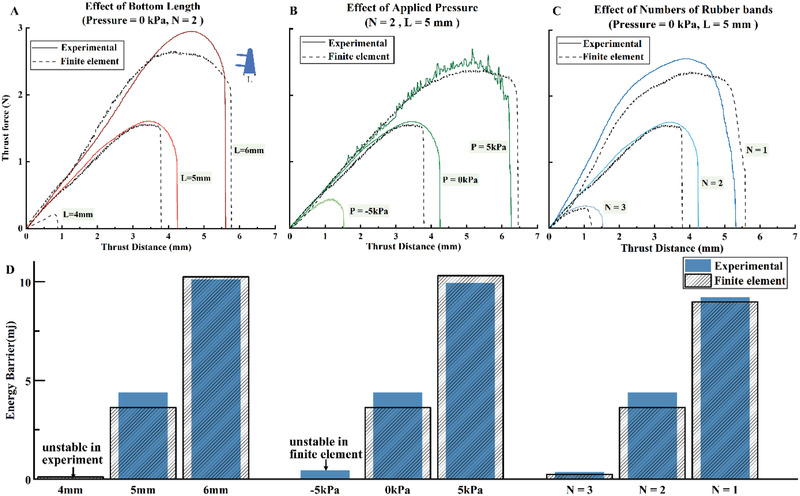
Finite element prediction and experimental validation for HSG triggering structures. A) The bottom width of the trigger block has a significant influence on the trigger sensitivity, which means that HSGs with different sensitivities can be designed by changing the bottom length of the trigger block. B) The use of negative pressure can significantly reduce the pushing force and distance required for triggering, while the use of positive pressure is the opposite. This means that the sensitivity of HSG can be controlled onboard. The curve fluctuations in the experimental results are caused by the adjustment of the air pressure in the air pressure control loop. C) The numbers of the rubber bands were varied to change the force applied to both ends of the HSG. N indicates the number of rubber bands used on one side. Experimental results presented in A–C) used one single‐joint HSG of each configuration (sample size *n* = 1). D) The trigger energy barrier is calculated from the total thrust work of finite element models and experimental data.

To evaluate the accuracy of the finite element models, the experimental characterization of the HSG triggered by a push block was conducted. To accurately measure the thrust forces and simplify the finite element models, the trigger hairs of the HSG in both experiment and element models were removed. Thrust force and distance were recorded, and finite element predictions were compared to the experimental data (**Figure** **2**A–C). The finite element models predicted experimental results with exceptional accuracy. Figure 2D shows the trigger energy barrier calculated from the total thrust work of finite element models and experimental data. Not only the sensitivity of the HSG can be adjusted in the design and production by changing the size of the trigger blocks and the number of rubber bands, but also by changing the air pressure without reproduction. We used a high‐speed camera to record the reflex process of the HSG under different inner pressure as shown in Movie S3 (Supporting Information) and the angle changing of the HSG during the trigger process is shown in Figure S2 (Supporting Information). Negative pressure applied in the air chamber makes the triggering quicker and easier, while positive pressure makes it slower and harder. With the pressure of −5, 0, and 5 kPa being applied to the internal chamber, the reflex time of the HSG is 30, 36, and 42 ms, respectively. When the pressure increases to 15 kPa, pushing the trigger hairs would not trigger the bending action.

### 2.3 Activate Bistable Snap‐Through

**Figure 3:**
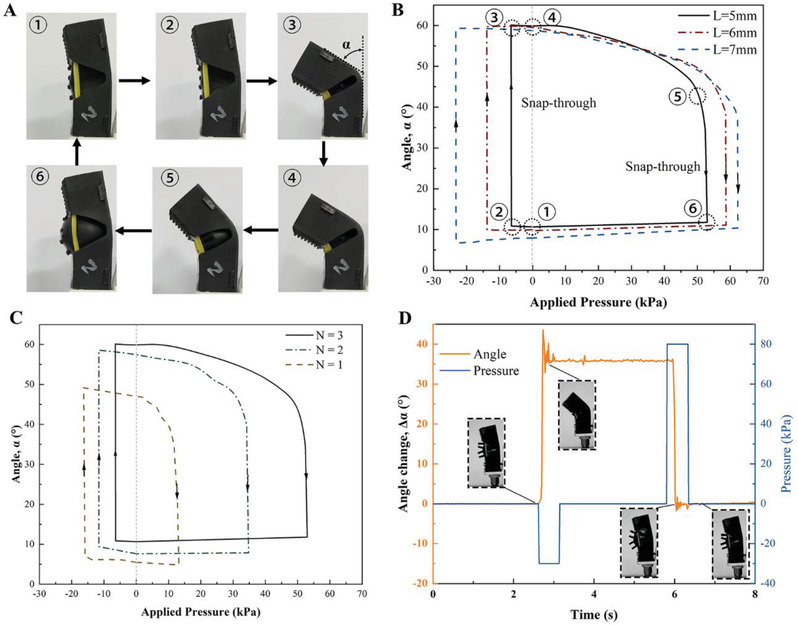
Active bistable snap‐through for HSGs with different design parameters under negative and positive pressure. A) The bending and straightening process of the one‐joint HSG (*L* = 5 mm, *N* = 3) upon the pressure changing. B) The bending angle changes with the pressure using different bottom lengths *L*. Two snap‐through regions are denoted with arrows in the curves. The numbers 1–6 marked in the curve correspond to the states 1–6 in A). C) The bending angle changes with the pressure using different numbers of rubber bands on each side. D) The HSG angle change under a falling edge and a rising edge of the pressure is captured by a high‐speed camera. Experimental results presented in B–D) used one single‐joint HSG of each configuration (sample size *n* = 1).

The HSG imitates the activating strategy of spider joints, using muscles to flex the joints and fluid pressure to straighten the joints. Further, negative pressure can also trigger the HSG, so that it can bend without external mechanical triggering. **Figure** **3**A shows that the bending and straightening process of the one‐joint HSG (*L* = 5 mm, *N* = 3) upon the pressure changing.

We recorded the change of HSG bending angle *α* upon different air pressure. The bottom length of the trigger blocks (denoted as *L*) and the number of the rubber bands on each side (denoted as *N*) were varied to quantify how critical design inputs affected pressure‐related performance (Figure 3B,C). When negative pressure is applied in the straightened state, the HSG would snap through directly to the bending state. When positive pressure is applied in the bending state, and before the positive pressure snap‐through occurs, there is a considerable region that can adjust the bending angle of the HSG until it reaches a specific pressure. When the length of the bottom surface of the trigger block increases, the minimum vacuum pressure values of the bending and straightening snap‐through increase. When the number of rubber bands is reduced, which means the squeezing forces on the trigger blocks decreases, higher vacuum pressure is required to produce bending snap‐through, while a lower positive pressure is required to produce straightening snap‐through.

Due to the bistable characteristics of the HSG, only a falling edge and a rising edge of air pressure are needed to activate the bending and straightening of the HSG, instead of continuous pressure input to hold the state. Movie S4 (Supporting Information) shows the bending and straightening process, and the bending angle changes Δ*α* and applied pressure were recorded as shown in Figure 3D. After state transition, the HSG can be maintained in another state without continuing to input pressure, which means that after the grasping action is completed, the HSG can maintain the grasping state without additional energy input.

### 2.4 Demonstration of Multimode Gripping

**Figure 4:**
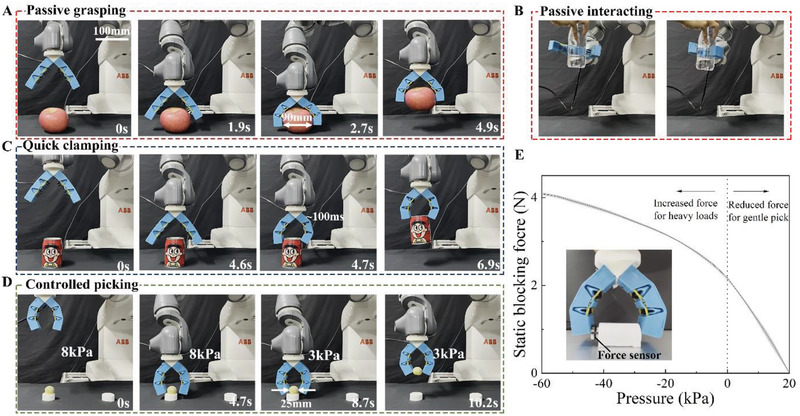
Multiple gripping strategies of the HSG. A) Leveraging the stress‐response strategy, the HSG automatically gripped the apple when they contacted. B) The HSG perceived and caught a bottle of water handed over by people, without the need for electronic component‐based sensors. C) The HSG grabbed a can before contact under the trigger of negative pressure, and the grabbing process was completed within 100 ms. D) Through the antagonism between the air pressure and the rubber bands, a cooked egg yolk is gently picked up without crushing. The blocking force can be controlled by the combined action of air pressure and rubber bands as shown in E). E) Static blocking force versus air pressure curves for the two‐joint HSG. The data are presented as the mean ± SD of five independent experiments (*n* = 5). The solid line corresponds to the mean values from five tested samples, while the shadow areas show the standard error of the mean.

The HSG can be readily integrated with the existing robotic platform. This section demonstrates its unique multiple gripping strategies enabled by the stress‐response strategy:

*Passive grasping and interacting*: Previous common grasping processes usually include three steps. First, the robot arm drives the gripper to move to the estimated position and stop. Second, the gripper moves or deforms to grab the object while the arm is stopped. Third, the gripper and the object are moved to the designated position by the arm. There is a noticeable pause between the first and second steps, the grippers usually work when the arm is stopped. However, when humans grasp an object, even without looking, the human hand can grasp the object as soon as it touches the object without stopping. The HSG achieves a similar function, as shown in **Figure** **4**A, leveraging its stress‐response strategy, the robotic arm can directly move onto the object without stopping. The grasping process would be automatically triggered when the object touched the trigger hairs. And for the intrinsic softness of HSG, the collision would not cause any damage to HSG or the object. This passive feature can also be applied to human–computer interaction. As shown in Figure 4B, the robot does not need sensing or predicting human actions and can quickly grab the bottle handed. Movie S5 (Supporting Information) shows the complete process of passive grasping, as well as the clamping process of other objects, and Movie S6 (Supporting Information) shows the complete process of passive interacting.

*Quick clamping*: The HSG can be triggered by negative pressure, which means that the grasping action can also be generated at the required position without touching the object. This grasping process can be accomplished within 100 ms through a pulse of negative pressure applied to the air chamber. This grabbing method is effective for situations where the trigger hairs cannot or does not want to be touched. Figure 4C demonstrates the Quick clamping function. Movie S7 (Supporting Information) shows the complete process of this clamping, as well as the clamping process of other objects.

*Controlled picking*: The rubber bands of HSG make it tend to bend, while the inflated air chamber tends HSG to straighten. The antagonism between the two can make the HSG produce a small grasping force for the picking of fragile objects. At the same time, due to the continuous action of the two antagonistic forces, it is like the human antagonistic muscles (such as the biceps and triceps) exerting force at the same time, which allows the HSGs to generate a small grasping force while increasing their stiffness and improving grasp stability. Figure 4D demonstrates the HSGs gently picking up a cooked egg yolk. In addition, the air pressure applied to the airbag can not only reduce the grasping force and realize gentle picking but also use negative pressure to increase the grasping force for heavy objects. The output force of HSG is related to the application of others, as shown in Figure 4E. Movie S8 (Supporting Information) demonstrates different kinds of Controlled picking.

## 3 Conclusion

The stress‐response strategy is a basic strategy of creatures. Some creatures (e.g., humans) have evolved advanced brain‐based decision‐making systems, but they still retain this underlying strategy, like hand dorsal reflex, eye blink reflex, and knee jerk reflex. This kind of reflex bypasses the brain and has a faster reaction speed than brain‐based movement, which can often help humans and animals to cope with emergencies under certain circumstances. Even plants, such as the Venus flytrap, use this stress‐response strategy to prey on insects. The evolution process has not eliminated this underlying mechanism, because it allows creatures to better respond to emergencies, and on the other hand, it reduces the burden of decision‐making on the brain.

The stress‐response strategy can be seen in some simple artificial systems, such as traps and thermal circuit breakers. It is difficult to predict when a beast will step on the trap or when a short circuit will occur, but this response to the environment can cope well with various emergencies, just like the stress‐response strategy of the creatures. However, it is worth thinking that as high‐level advanced artificial systems, robots, are rarely leveraging this stress‐response strategy, especially for the end actuators. In the process of robot design, most emphasize the absolute control of the end actuators. The information must go through a process from sensors to computing units, and then to actuators. For complex actions, this process can ensure that everything is under control, but it also leads to slow speed when performing some simple actions.

The HSG explored the application of the stress‐response strategy in robotic systems. We presented the design and development of a soft robotic gripper leveraging the stress‐response strategy for high‐speed dynamic grasping. The first major contribution was the mechanical instability embedded in the HSG enables fast perception and actuating process for high‐speed dynamic grasping. The second major contribution was the pneumatic control system inspired by spiders enabling functions including 1) recovery after the trigger; 2) actively generate triggers; 3) control the sensitivity of triggers; 4) increase or decrease gripping force to cope with different objects. While introducing the stress‐response strategy, the HSG can still be precisely controlled. Our approach offers a new paradigm for designing practical high‐speed soft robotic systems.

## 4 Experimental Section

### The HSG Fabrication

All the components required for making the HSG are shown in Figure S1 (Supporting Information). A three‐components polyurethane system (8400, Hei‐Cast) was used as the elastomeric material for the main part and the trigger blocks of the HSG. The production process adopts molding and demolding approaches. The main part uses the mixing ratios in A: B: C = 100: 100: 400 and the trigger blocks use the mixing ratios in A: B: C = 100: 100: 150. The rubber bands are commercially available with a 1.5 mm × 1.5 mm square cross‐section area and 25 mm perimeter. The fixing fixers of the rubber bands inside the HSG were obtained by mechanical processing using Polycarbonate material.

### Materials Characterization

Uniaxial tensile tests were conducted to characterize the material properties of polyurethane materials and rubber bands, using the tensile pressure testing machine (ZQ‐990B, ZHIQU). For the polyurethane materials, five samples of each different mixing ratio (A: B: C = 100: 100: 400 and A: B: C = 100: 100: 150) were stretched to failure at a rate of 120 mm s^−1^. The stress–strain curve of the different mixing ratios is shown in Figure S3 (Supporting Information). For the rubber bands, seven samples were stretched to failure at a rate of 120 mm s^−1^. The tensile strain to failure and cyclic tensile loading and unloading performance are shown in Figure S4 (Supporting Information). The prestretching strain of the rubber bands used in the HSG is about 160%.

### FEA Simulation

The 3D models of the HSG were built by Solidworks software and the commercial FEA software Abaqus/CAE was used for analysis, employing the Abaqus/Explicit solver. The geometry of all parts was imported into Abaqus CAE as an x_t file. The main body and the trigger blocks are meshed with 4‐node linear tetrahedron elements (C3D4) and model as Mooney–Rivlin two‐parameter model. In the model, C10 = 0.218 490 375, C01 = −0.0 374 194 854, D1 = 0 for main body and C10 = 0.347 770 636, C01 = −0.08 076 209 326, D1 = 0 for trigger blocks. The rubber bands are meshed with 2‐node linear 3D truss elements (T2D3) and modeled as the Mooney–Rivlin two‐parameter model with C10 = 0.218 490 375, C01 = −0.0 374 194 854, D1 = 0 for the prestressed process. The push pads and fixers of rubber bands are modeled as 3D discrete rigid elements. In the simulation process, due to the limitation of computing power, the moving speed of pushing pads is set at 2 mm s^−1^.

### External Trigger Experiment

The HSG was fixed on a 3D‐printed supporter. The screw motor platform was used to drive a dynamometer to touch the contact position of the two trigger blocks at a speed of 0.1 mm s^−1^ and record the change of contact force during the movement. The air pressure is measured by a pressure sensor (DP‐101A, Panasonic Corp.) installed at the inlet of the HSG.

### Dynamic Response Measurement

A high‐speed camera (V2512, PHANTOM) at a sampling rate of 3000 Hz was used to track the motion of the whiskers. And the rotation angle is captured by free video analysis and modeling tool (Tracker 5.1.3).

### Grasping Experiment

To demonstrate the application of the HSG application grasping process, the HSG on the end of a dual‐arm robot (IRB14000, ABB) for the grasping test was installed. The connecting piece at the connection place is made by 3D printing, and the end of the connecting piece has a tracheal interface for adjusting the air pressure applied to the cavity inside the HSG.

### Statistical Analysis

No filtering was applied in the experimental results presented in this work. For measuring thrust force versus thrust distance, experimental results presented used one single‐joint HSG of each configuration (sample size *n* = 1). For measuring bending angle under the applied pressure, the angle was captured by the point track function of Phantom Camera Control Application (version 2.7), and experimental results presented used *n* = 1. For measuring blocking force versus applied pressure of the two‐joint HSG, the data are presented as the mean ± SD of five independent experiments (*n* = 5). For measuring the stress–strain curves of the polyurethane materials used to fabricate the HSG, five samples of different mixing ratios are tested (*n* = 5), and the data are presented as the independent curves. For measuring the tensile strain performance of the rubber bands, seven samples (*n* = 7) are tested, and the data are presented as independent curves. For measuring cyclic tensile loading and unloading performance of rubber bands, one rubber band (*n* = 1) is used for each maximum stretch rate, and the data of 50 cycles are presented.

## Conflict of Interest

The authors declare no conflict of interest.

## Supporting information

Supporting Information

Supplemental Movie 1

Supplemental Movie 2

Supplemental Movie 3

Supplemental Movie 4

Supplemental Movie 5

Supplemental Movie 6

Supplemental Movie 7

Supplemental Movie 8

## Data Availability

The data that support the findings of this study are available from the corresponding author upon reasonable request.
