# Supplementary material for: A Bioinspired Stress‐Response Strategy for High‐Speed Soft Grippers
Source: Adv Sci (Weinh). 2021 Sep 2;8(21):2102539. doi: 10.1002/advs.202102539 (PMC8564422; doi:10.1002/advs.202102539)
Supplement: Supplementary file 1 — Supporting Information [file ADVS-8-2102539-s003.pdf]

WILEY-VCH

© 2021 Wiley-VCH GmbH

ADVANCED SCIENCE
Open Access

Supporting Information

for *Adv. Sci.,* DOI: 10.1002/advs.202102539

A bio-inspired Stress-response Strategy for High-speed Soft Grippers

*Yangqiao Lin, Chao Zhang, Wei Tang, Zhongdong Jiao, Jinrong Wang, Wei Wang, Yiding Zhong, Pingan Zhu, Yu Hu, Huayong Yang, and Jun Zou**

Supporting Information

A bio-inspired stress-response strategy for high-speed soft grippers

*Yangqiao Lin, Chao Zhang, Wei Tang, Zhongdong Jiao, Jinrong Wang, Wei Wang, Yiding Zhong, Pingan Zhu, Yu Hu, Huayong Yang, and Jun Zou**

Contents:

Figures:
Figure S1. All parts used to assemble the HSG.
Figure S2. The dynamic response of the bending angle when HSG is externally triggered.
Figure S3. The stress-strain curves of the polyurethane materials used to fabricate the HSG.
Figure S4. Strain permanence of the rubber bands.

Supplementary Movies:
Supplementary Movie 1: Catching a thrown baseball.
Supplementary Movie 2: Finite element simulation and experimental validation.
Supplementary Movie 3: Control trigger sensitivity by applied pressure.
Supplementary Movie 4: Active bistable snap-through.
Supplementary Movie 5: Passive gripping demonstration.
Supplementary Movie 6: Passive interacting demonstration.
Supplementary Movie 7: Quick clamping demonstration.
Supplementary Movie 8: Controlled picking demonstration.

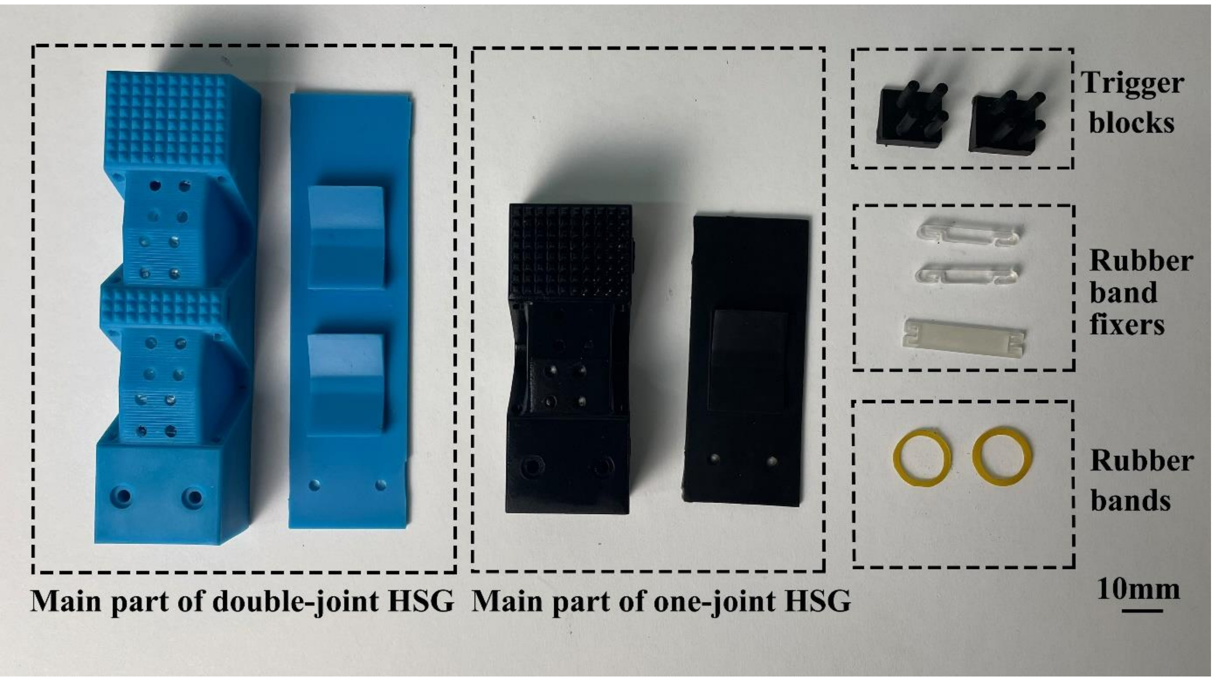

Figure S1. All parts used to assemble the HSGs. The main part of double-joint and one-joint HSGs use the same mixing ratios of polyurethane system (8400, Hei-Cast) and different pigments for toning.

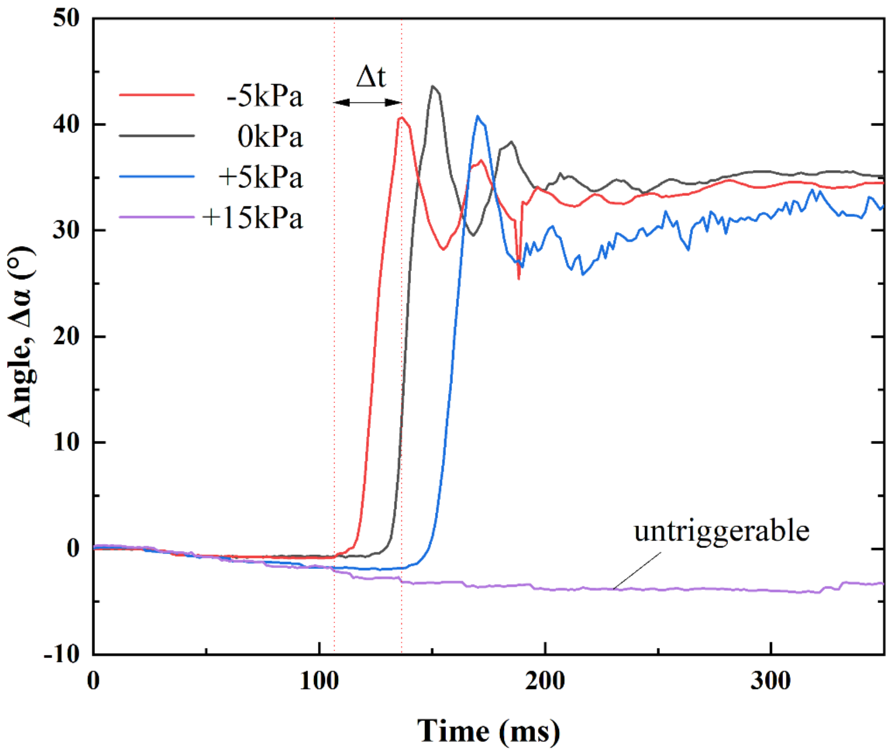

Figure S2. The dynamic response of the bending angle when HSG is externally triggered. The Δt marked in the figure represents the reflex time of bending from the minimal angle to the maximal angle. With the inner chamber pressure of -5 kPa, 0 kPa, and 5 kPa being applied to the internal chamber, the reflex time of the HSG is 30 ms, 36 ms, and 42 ms, respectively. When the inner chamber pressure is increased to 15 kPa, pushing the trigger hairs cannot make the HSG triggered. The complete comparison process can be seen in Supplementary Movie S3.

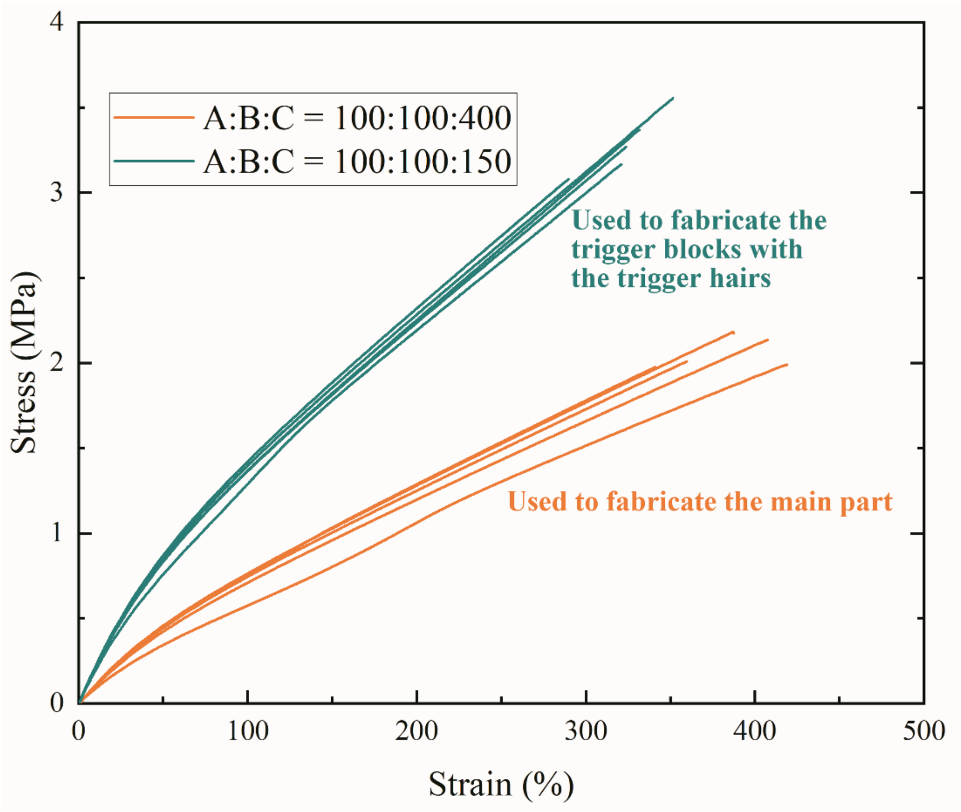

Figure S3. The stress-strain curves of the polyurethane materials used to fabricate the HSG. Hei-Cast 8400 is three-component polyurethane system and used to fabricate the trigger blocks and the main body of HSG. The mixing ratios of the trigger blocks and the main body are 100: 100: 150 and 100: 100: 400, respectively. Tensile strain performance, to failure, is for five samples of different mixing ratios.

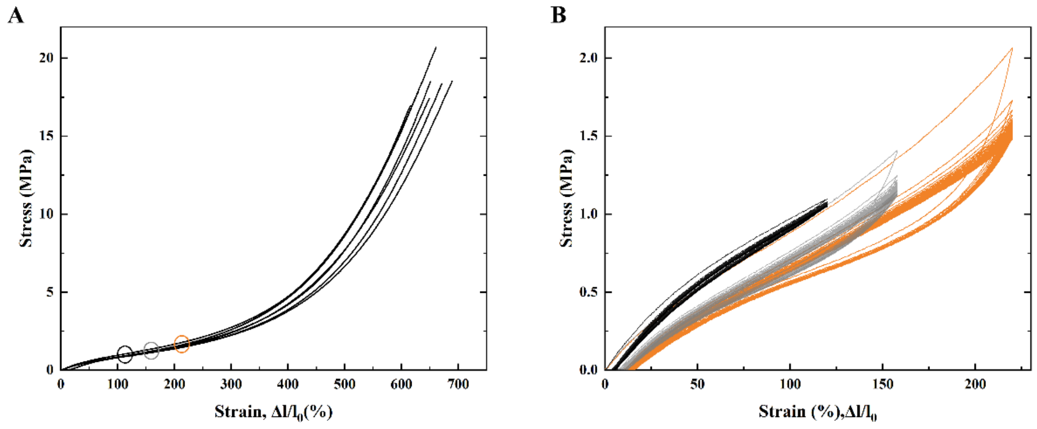

Figure S4. Strain performanence of the rubber bands. (A) Tensile strain performance, to failure, for seven samples of the rubber bands. (B) Cyclic tensile loading and unloading performance of rubber bands at strains corresponding to the colored circles in (A).
